# Phase 2A Learnings Incorporated into RewinD-LB, a Phase 2B Clinical Trial of Neflamapimod in Dementia with Lewy Bodies

**DOI:** 10.14283/jpad.2024.36

**Published:** 2024-02-09

**Authors:** N. D. Prins, W. de Haan, A. Gardner, K. Blackburn, H.-M. Chu, J. E. Galvin, John J. Alam

**Affiliations:** 1 Brain Research Center, Amsterdam, Netherlands; 2 Amsterdam UMC, Amsterdam, Netherlands; 3 CervoMed (formerly EIP Pharma) Inc, 20 Park Plaza, Suite 424, Boston, MA 02116 USA; 4 Anoixis Corporation, Natick, USA; 5 University of Miami Miller School of Medicine, Boca Raton, USA

**Keywords:** Neflamapimod, dementia with Lewy bodies (DLB), clinical trial, p38 MAPK

## Abstract

**Background:**

In an exploratory 91-participant phase 2a clinical trial (AscenD-LB, NCT04001517) in dementia with Lewy bodies (DLB), neflamapimod showed improvement over placebo on multiple clinical endpoints. To confirm those results, a phase 2b clinical study (RewinD-LB, NCT05869669 ) that is similar to AscenD-LB has been initiated.

**Objectives:**

To optimize the choice of patient population, primary endpoint, and biomarker evaluations in RewinD-LB.

**Design:**

Evaluation of the efficacy results from AscenD-LB, the main results of which, and a re-analysis after stratification for absence or presence of AD co-pathology (assessed by plasma ptau181), have been published. In addition, the MRI data from a prior phase 2a clinical trial in Early Alzheimer’s disease (AD), were reviewed.

**Setting:**

22 clinical sites in the US and 2 in the Netherlands.

**Participants:**

Probable DLB by consensus criteria and abnormal dopamine uptake by DaTscan™ (Ioflupane I123 SPECT).

**Intervention:**

Neflamapimod 40mg capsules or matching placebo capsules, twice-a-day (BID) or three-times-a-day (TID), for 16 weeks.

**Measurements:**

6-test Neuropsychological Test Battery (NTB) assessing attention and executive function, Clinical Dementia Rating Sum-of-Boxes (CDR-SB), Timed Up and Go (TUG), International Shopping List Test (ISLT).

**Results:**

Within AscenD-LB, patients without evidence of AD co-pathology exhibited a neflamapimod treatment effect that was greater than that in the overall population and substantial (cohen’s d effect size vs. placebo ≥ for CDR-SB, TUG, Attention and ISLT-recognition). In addition, the CDR-SB and TUG performed better than the cognitive tests to demonstrate neflamapimod treatment effect in comparison to placebo. Further, clinical trial simulations indicate with 160-patients (randomized 1:1), RewinD-LB conducted in patients without AD co-pathology has >95% (approaching 100%) statistical power to detect significant improvement over placebo on the CDR-SB. Preliminary evidence of positive treatment effects on beta functional connectivity by EEG and basal forebrain atrophy by MRI were obtained in AscenD-LB and the Early AD study, respectively.

**Conclusion:**

In addition to use of a single dose regimen of neflamapimod (40mg TID), key distinctions between phase 2b and phase 2a include RewinD-LB (1) excluding patients with AD co-pathology, (2) having CDR-SB as the primary endpoint, and (3) having MRI studies to evaluate effects on basal forebrain atrophy.

## Introduction

**D**ementia with Lewy bodies (DLB) is the 3rd most common chronic neurodegenerative disease (1), after Alzheimer’s disease (AD) and Parkinson’s disease (PD). Patients with DLB have deficits in both cognition and motor function (2, 3), and they incur a greater rate of cognitive decline (4), higher healthcare costs, report lower quality of life, and have caregivers with higher levels of distress compared to patients with AD (5, 6). Despite the clinical implications and there being no therapies specifically approved to treat DLB, there have been only limited drug development efforts directed against DLB (7). This is attributable to, among other factors, a lack of consensus on clinical endpoints, a lack of DLB-specific biomarkers, and until recently a lack of diagnostic criteria for clinical trials. Within that context, the recent publication of the AscenD-LB study (8) represents the first report of a positive phase 2 clinical trial with a drug that is targeting the underlying disease process (i.e., a potentially disease-modifying drug). In that exploratory, placebo-controlled 16-week treatment phase 2a clinical study, neflamapimod, an oral drug targeting the effects of neuroinflammation on the molecular mechanisms underlying degeneration of cholinergic degeneration in the basal forebrain (9, 10), was evaluated for treatment effects in patients with DLB against a range of clinical endpoints. In this study, neflamapimod reduced dementia severity (assessed by Clinical Dementia Rating Sum-of-Boxes, CDR-SB) and improved functional mobility (assessed by Timed Up and Go, TUG, test), in comparison to placebo treatment. In addition, at the highest dose studied (40mg three-times-daily, TID; 40mg twice-a-day, BID, was also evaluated in the study), neflamapimod improved outcomes in a six-test cognitive test battery, with the greatest benefits observed for tests of attention. Further analyses of the AscenD-LB study, specifically results stratified by pretreatment levels of phosphorylated tau as a marker of Alzheimer’s disease (AD) related co-pathology that showed the effects of neflamapimod are most prominent in the patients with pure DLB (i.e., patients without AD co-pathology), have also been recently reported (11). As an exploratory study, the AscenD-LB study did not have a primary hypothesis, and therefore definitive conclusions regarding efficacy could not be made. Rather, to confirm the phase 2a results and demonstrate proof-of-concept, a phase 2b clinical study (RewinD-LB) that is similar in design to AscenD-LB has been initiated. One major difference between AscenD-LB and RewinD-LB is the use of a single dose regimen, 40mg TID, in RewinD-LB; a decision based on 40mg BID being largely ineffectual in AscenD-LB, while 40mg TID demonstrated clinical activity against a range of clinical endpoints (10). Other differences in study design include the choice of patient population, the primary endpoint, and biomarker evaluations. The objective of the current report is to detail the major learnings from AscenD-LB and from a prior neflamapimod phase 2a study in Alzheimer’s disease (AD) (12), that drove the decision to implement these additional changes (i.e. changes in design other than the decision on dose); learnings that may also have application to clinical trials of other novel agents for the treatment of DLB. In addition, we report on the impact of the changes in design between the two studies on the statistical power of the RewinD-LB to meet its primary objective of demonstrating a significant effect, relative to placebo, of neflamapimod treatment on change in CDR-SB (i.e., to demonstrate proof-of-concept for neflamapimod as a treatment for DLB).

## Methods

The AscenD-LB study, the results of which have been reported (8), was a 91-patient, 16-week, doubleblind placebo-controlled phase 2a clinical study in patients with (Mini-Mental Status Examination, MMSE, between 16 and 28) probable DLB by 2017 consensus criteria [dementia, with at least one core clinical feature of DLB and demonstrated abnormality in dopamine uptake by DaTscan™ (Ioflupane I123 SPECT)], receiving cholinesterase inhibitor therapy (>3 months, stable dose for >6 weeks), and having Mini-Mental Status Examination (MMSE) score between 16 and 28.

To determine whether neflamapimod was clinically active in DLB against one or more aspects of the disease, the effects of neflamapimod relative placebo were evaluated against a range of clinical outcome measures. The designated primary outcome measure of the study was a study-specific six-test neuropsychological test battery (NTB) and the major secondary outcome measure was the Clinical Dementia Rating Scale Sum of Boxes (CDR-SB). The NTB was designed to assess the cognitive domains most impacted in DLB: attention (Identification, Detection tests), executive function (Category Fluency, Letter Fluency, One Back accuracy), and visual learning (One Card Learning); and the CDR-SB assesses both cognition and function (i.e., dementia severity) through scoring six domains (memory, orientation, judgement & problem solving, community affairs, home & hobbies, and personal care) on a scale of 0–3 (total range 0–18, higher scores indicating worse dementia). Additional secondary outcome measures were memory function (assessed by International Shopping List Test (ISLT), and motor function (specifically, functional mobility, assessed by the Timed Up and Go, TUG, test). The ISLT is a validated 12-word verbal list learning test that reports out three scores: Immediate Recall, (scored 0–36, the sum of three immediate recall trials), Delayed Recall (0–12, single recall, 20 to 25 min after initial trials), and Recognition (0–12, accurate recognition of the words in the original trials). The TUG test, measuring functional mobility, monitors the time in seconds that a subject takes to rise from a chair, walk three meters, turn around 180 degrees, walk back to the chair, and sit down while turning 180 degrees. The NTB and ISLT were performed at baseline and at weeks 4, 8, and 12 during treatment. The CDR-SB and TUG test were performed at baseline and at weeks 8 and 16. The Mini Mental Status Examination (MMSE) was also an outcome measure of the study. However, evaluation of the effects on the MMSE were limited by COVID-19 pandemic restrictions, which led to a third of on-study evaluations either being missed or conducted remotely via video, an approach that has not been validated. As such, the on-treatment MMSE data were not evaluated in the main reporting of study results.

Patients in the AscenD-LB study were randomized 1:1 to either neflamapimod (NFMD) 40mg capsules or matching placebo and then, based on body weight, assigned to either a twice-daily (BID) [weight<80 kg; 40mg BID neflamapimod or placebo BID] or thrice-daily (TID) [weight≥80 kg; 40mg TID neflamapimod or placebo TID] regimen.

After completion of the AscenD-LB study and reporting of the primary results (which did not differentiate by AD co-pathology status), pre-treatment plasma ptau181 levels were determined on the Simoa HD-X platform (Quanterix Inc., Billerica, MA) and participants were then grouped based on a cut-off for AD pathology of 2.2 pg/mL (established in a separate cohort to identify AD from healthy controls). The clinical outcomes for patients given placebo and those treated with neflamapimod 40mg TID (the more clinically active of the two doses studied) within each sub-group (baseline plasma ptau181 < and ≥2.2 pg/mL). The results of this stratified analysis have been published recently (11) and separately from the primary publication.

The only biomarker evaluated in the AscenD-LB study was task-free, eyes-closed Electroencephalography (EEG), obtained in accordance with the 10–20 International System of Electrode placement. Due to restrictions on clinical research activities imposed by COVID-19, baseline and week 16 EEG assessments were available in 29 of 91 patients enrolled; 17 receiving placebo, and 6 each in neflamapimod 40mg BID and 40mg TID. EEG data were processed and analyzed centrally at the VU Medical Center in Amsterdam. From each EEG, artefact-free epochs were visually selected by an experienced neurophysiologist (W. de Haan), blinded to the treatment. Quantitative EEG analysis was divided into 2 parts: (1) spectral analysis, including relative power spectral density in commonly used frequency bands and posterior dominant peak frequency, and (2) functional connectivity analysis, specifically corrected Amplitude Envelope Correlation (AECc) (14) to assess interregional communication. 2-sided Wilcoxon Rank Sum Test p-values are reported.

Because of the emergence of basal forebrain (specifically nucleus basalis of Meynert, NbM) volumetry by MRI has emerged recently as a treatment biomarker, and MRI studies were not performed in AscenD-LB, the MRI data from a prior phase 2a study (NCT024231220) (12) in Early AD were evaluated for the potential of NbM atrophy by MRI to be included in the RewinD-LB. This other study was a blinded comparison of neflamapimod 40mg BID vs 125 mg BID, dosed for 12 weeks in patients with amyloid-PET documented early AD (MMSE 20–28). Structural (3D T1 Isotropic) MRI and resting-state fMRI (TR=2225, 200 volumes) were obtained at baseline and end-of-treatment (n=13 participants). In the retrospective analysis, NbM volume and functional connectivity of the NbM were assessed at Amsterdam UMC as recently described (15).

Registrations on clinicaltrials.gov: NCT04001517 (AscenD-LB), NCT05869669 (RewinD-LB), NCT02423122 (Phase 2a in Early AD).

## Results

### Patient Population

Table 1 gives the disease characteristics at baseline and the results of cognitive testing at study entry in the for the patients enrolled in AscenD-LB. With the use of the current consensus clinical criteria for probable DLB, there is a high prevalence of each of the core disease features of DLB [fluctuating cognition (61%), visual hallucinations (58%), REM sleep disorder (66%), parkinsonism (81%)] and 85% of patients exhibiting ≥ 2 features. Further, in the cognitive testing utilizing the NTB the most severe deficit (mean 2.6 SD lower score than the age-adjusted adjusted norm) was in the One Back (accuracy) test, a test of attention and working memory; and the two tests of attention (Identification, Detection), each showed a mean 1.6 SD lower performance vs. age-adjusted norm. That is, the patients enrolled exhibited demonstrable and significant attentional deficit associated with DLB. The other domain generally considered to be associated with DLB is executive function. We assessed executive function using the Letter Fluency (−0.7 SD) and Category Fluency (−1.0 SD) and found that scores were less impacted. This is likely due to the patients being predominantly early stage DLB (37% CDR 0.5, 52% CDR 1.0), who are known to have lesser deficits in executive function than patients with moderate and severe DLB (16). Of note, in meeting the consensus criteria for probable DLB, patients had to have on a clinical basis dementia, i.e., “progressive cognitive decline of sufficient magnitude to interfere with normal social or occupational functions, or with usual daily activities” (13). As such, the patients with CDR 0.5 in the study were patients with very mild dementia rather than MCI-LB (also known as prodromal DLB) which requires that the patient have preserved or minimally impacted functional abilities that do not meet the criteria for dementia (17).

**Table 1:** Patient Population Enrolled in AscenD-LB Study

**Baseline Disease Characteristics (N=91)**		**Baseline z-scores**^1^ **on the individual tests within NTB**	
CDR 0.5/1.0/2.0	37%/52%/11%	Identification	−1.6 (1.6)
MMSE	23.0 (3.6)	Detection	−1.6 (1.6)
Fluctuating cognition	61%	One card learning	−1.1 (0.8)
Visual hallucinations	58%	One back (accuracy)	−2.6 (1.6)
REM sleep disorder	66%	Letter Fluency	−0.7 (1.0)
Parkinsonism	81%	Category Fluency	−1.0 (1.4)
≥2 features	85%		

Mean (SD), except where % of patients randomized is shown. 1. z-scores relative to age adjusted norm; NTB — Neuropsychological Test Battery consisting of the six individual tests on the right hand side of the table.

In order to further define the optimal patient population to study in future studies, as pre-specified in the AscenD-LB protocol the AscenD-LB data were re-analyzed on a post-hoc basis following stratification of the sample according to the presence of Alzheimer’s disease (AD) co-pathology at baseline, as classified by pre-treatment levels of plasma ptau181. The motivation for that analysis, which was recently published (11), was a report published after the primary study was completed that showed that plasma ptau181 can be assessed in patients with DLB to identify patients with AD related co-pathology (18), which is associated with more advanced disease, including greater levels of neurodegeneration (19). That stratified analysis showed that the beneficial effects of neflamapimod compared to placebo over 16 weeks treatment were greater in those patients with normal pre-treatment levels of plasma ptau181, with treatment effect sizes of 0.7 or higher, for each of CDR-SB, TUG, attention, and ISLT-recognition. In Table 2, we have extracted the results from both the primary publication (8) and the publication with the stratified results (11), and show the results in the neflamapimod 40mg TID patients compared to placebo in the Ascend-LB study for the overall patient population that includes a mixed population with respect to AD co-pathology (46% with, 54% without), and for those patients without AD co-pathology (i.e., excluding patients with plasma ptau 181 greater than the cut-off). With exclusion of patients with AD co-pathology, the magnitude of the level of improvement compared to placebo with neflamapimod treatment, assessed by Cohen’s d effect size, is substantially higher for all the parameters. In addition, there is significant improvement compared to placebo on ISLT-recognition and near significance for ISLT total score, effects that were not seen in the overall patient population. With these findings, and in order to be able to demonstrate the most robust proof-of-concept, patients with AD co-pathology (as assessed by plasma ptau181) will be excluded in the phase 2b study. In order to reduce screen failures due to not meeting the plasma ptau181 criteria, as all but one of the seven patients with CDR global score of 2.0 had elevated plasma ptau181 at baseline in AscenD-LB, only patients with a global CDR score of 0.5 or 1.0 (i.e., patients with Early DLB), will be enrolled. In addition, as these Early DLB patients may not be receiving cholinesterase inhibitor therapy, treatment-naïve patients will be eligible in RewinD-LB. Randomization will be stratified by whether participants are treatment naïve, receiving cholinesterase inhibitor therapy, or receiving therapy with both a cholinesterase inhibitor and memantine.

**Table 2:** AscenD-LB Results in Neflamapimod 40mg TID, in Overall Patient Population and in Patients without AD Co-Pathology

	**Overall Study Population**				**Patients Without AD Co-pathology (Plasma ptau181 < cutoff)**			
	**N=NFMD, Placebo**	**Difference**^1^ **(95% CI)**	**p-value**	**Cohen’s d Effect size**	**N= NFMD, Placebo**	**Difference**^1^ **(95% CI)**	**p-value**	**Cohen’s d Effect size**
NTB	19,37	+0.17 (0.00,0.35)	0.049	0.47	11,19	+0.21 (−0.07,0.49)	0.13	0.56
Attention	19,36	+0.28 (0.04,0.51)	0.023	0.41	11,18	+0.42 (0.07,0.78)	0.023	0.78
CDR-SB	20,38	−0.56 (−0.96,−0.16)	0.007	0.31	11,22	−0.60 (−1.04,−0.06)	0.031	0.74
TUG	20,38	−1.4 (−2.6,-0.2)	0.024	0.50	11,20	−3.1 (−4.7,−1.6)	<0.001	0.74
ISLT	20,42	+0.32 (−0.48,1.12)	NS	0.15	11,22	+2.1 (0.0,4.2)	0.053	0.55
ISLT - RECOG	19,39	+0.47 (−0.17,1.11)	0.15	0.17	10,21	+1.4 (0.02,2.5)	0.024	1.0

1. Difference between neflamapimod and placebo from mixed model for repeated measures (MMRM) analysis. Improvement reflected by negative sign for CDR-SB and TUG and positive sign for other measures. Abbreviations: NTB — Neuropsychological Test Battery; CDR-SB — Clinical Dementia Rating Sum of Boxes; TUG — Time Up and Go test; ISLT — International Shopping List Test; RECOG — Recognition

### Primary Endpoint

From both Table 2, as well the full analysis of phase 2a in our previous publication (10) the CDR-SB and the TUG perform better than the cognitive test battery, the NTB, to demonstrate neflamapimod treatment effect in comparison to placebo.

To further evaluate the study endpoints, sample size was evaluated by power analysis via simulations, conducted by utilizing the data in AscenD-LB for the major clinical endpoints in the neflamapimod 40mg TID and placebo groups, to generate for each patient a change from baseline for each endpoint at individual visits over the course of the simulated clinical study, and then analyzing the result of each clinical trial utilizing the linear mixed effects model for repeated measures (MMRM) that will be utilized to analyze the phase 2b study. Based on the simulation of 100 clinical trials with 80 patients per treatment group, and assuming a 10% dropout rate, there is ∼85% power with the NTB, 95% power with TUG, and >95% power with CDR-SB (approaching 100%) to detect a treatment effect at an alpha level of 0.05. Notably, the statistical power for the various endpoints is 10–15% lower when the study includes a DLB population that is mixed for the presence or absence of AD co-pathology.

### Biomarkers

The only biomarker evaluated in the AscenD-LB study was Electroencephalography (EEG). Task-free, eyes-closed EEG recordings, performed at Baseline and Week 16, were available in 29 of 91 patients enrolled (the remaining patients did not receive a week 16 EEG, primarily due to limitations imposed by COVID19 pandemic restrictions on clinical research). In the spectral analysis, globally comparing overall baseline with follow-up data, all treatment groups showed a modest trend towards improvement, though on most measures no neflamapimod-specific effects were discernible, including on dominant peak frequency. In the functional connectivity analysis, mean AECc (Amplitude Envelope Correlation to assess interregional communication) in the beta band was dose-dependently increased with neflamapimod: mean AECc beta was increased with neflamapimod TID versus all placebo (p=0.033 (2-sided Wilcoxon rank-sum test) and versus placebo TID (p=0.01). Across the electrodes, the increase in AECc beta was also significant (p<0.05) in the 14 of 20 electrodes utilizing FDR-corrected permutation t-tests. AECc in each of the other bands was stable or slightly improved with no treatment group differences.

Recently, basal forebrain atrophy on MRI has emerged as a potential biomarker for assessing drug effects for approaches targeting cholinergic degeneration. As MRI studies were not performed in the AscenD-LB study, MRI images pre-treatment and after 12 weeks’ treatment with neflamapimod from a previously completed phase 2a study in patients with early AD (n=15) were accessed and the volume of the nucleus basalis of Meynert (NbM), as well its functional connectivity was analyzed using validated techniques, as previously described (20). As reported recently at a scientific conference (21), the analysis demonstrated that the NbM volume was higher at the end of neflamapimod treatment (EOT, mean 3.1% higher vs. baseline, p=0.03, see Figure 2A); with 8 of 15 patients having greater than 3% NbM higher volume at EOT, compared to baseline. Treatment with neflamapimod was also associated with higher functional dynamic connectivity between the NbM and deep grey matter (DGM) at EOT (mean 11% higher vs. baseline, p=0.04, see Figure 2B); with 6 of 13 showing a greater than 10% higher dynamic NbM-DGM connectivity at EOT, compared to baseline.

**Figure 1:**
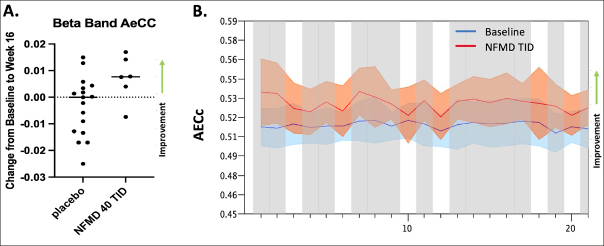
Neflamapimod improves beta functional connectivity on EEG (A) Corrected Amplitude Envelope Correlation (AECc, median & interquartile range are shown) in the beta band (13–30 Hz) significantly increased with neflamapimod TID (n=6) vs. all placebo (n=17, p=0.033) and vs. placebo TID (n=6, p=0.01). (B) Lower beta (13–20 Hz) AECc changes in the neflamapimod 40mg TID group. Grayscale: p<0.05, based on FDR-corrected permutation t-tests. Color bands represent 2 × SEM. X-axis represents EEG electrodes (10–20 system).

**Figure 2:**
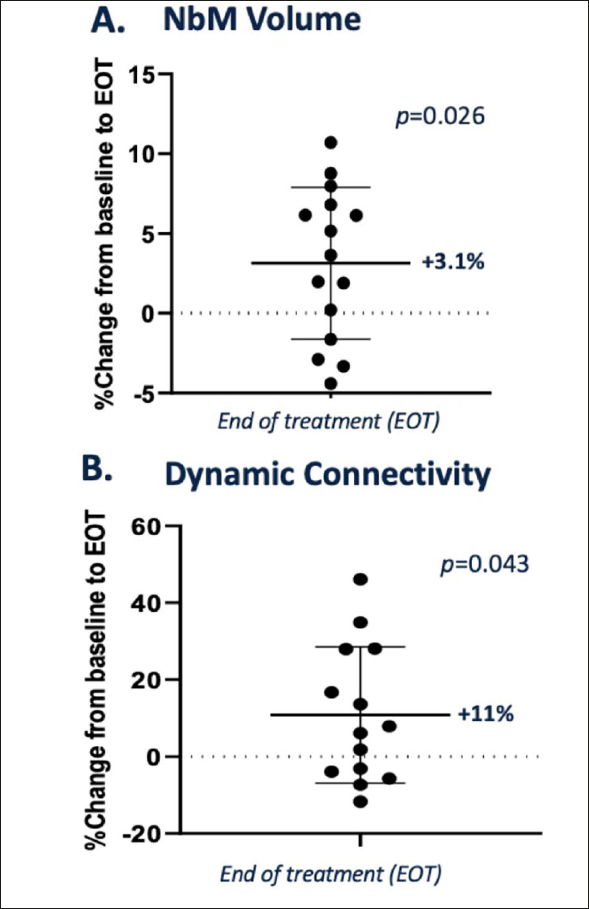
NEffects of 12 weeks neflamapimod treatment on the basal forebrain in patients with Early AD, assessed by MRI A. Nucleus of basalis of Meynert (NbM) volume. NbM volume was statistically significantly higher at the end of treatment compared to baseline (EOT, mean 3.1% higher vs. baseline, p=0.026). B. Functional dynamic connectivity. Neflamapimod was also associated with a statistically significantly higher functional dynamic connectivity between the NbM and deep grey matter (DGM) at EOT (mean 11% higher vs. baseline, p=0.04).

## Discussion

**Figure 3:**
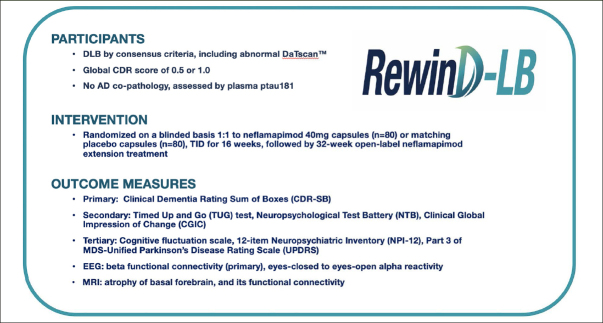
Overview of RewinD-LB Phase 2b study of neflamapimod in Early DLB.

In recent years there has been progress in diagnostic criteria for DLB for clinical and research purposes (13). This has led to advances in characterizing the presentation and progression of DLB. As testament to that progress, the AscenD-LB study was able to recruit a DLB patient population with demonstrated attentional deficits and obtained evidence of clinical activity of a potentially disease-modifying therapy. Utilizing the results of that study, both with respect to the neflamapimod treatment effect and the performance of various aspects of the clinical trial design, the RewinD-LB study was designed and is initiated to confirm the findings of the AscenD-LB study. The design of RewinD-LB (see Figure 3) replicates many aspects of AscenD-LB, e.g., it is also a 16-week placebo-controlled study. In particular, the inclusion criteria achieved its objectives of enrolling a cognitively impaired early stage DLB patient population. Accordingly, the eligibility criteria related to DLB definition will not be changed for the Phase 2b study. However, in other areas various learnings from the phase 2a studies support changes to the AscenD-LB study design. Specifically, key distinctions from phase 2a in the RewinD-LB study include the use of a single daily dose regimen of neflamapimod (40mg TID, based on the dose-response analysis of the study and on observations in AD studies (8)), choice of CDR-SB as primary endpoint, and the exclusion of patients with AD co-pathology (note: to enrich for such patients, the global CDR score at entry will be limited to 0.5 or 1.0; in AscenD-LB, 6 (86%) of 7 CDR=2.0 participants vs. 34 (44%) of 77 CDR=0.5 or 1.0 had elevated plasma ptau181, p=0.03) ).

With these modifications to the design, the sample size calculations the RewinD-LB phase 2b study has greater than 95% statistical power to meet its primary objective. In addition, in terms of biomarker evaluations, along with having EEG evaluations which had been included in AscenD-LB RewinD-LB adds MRI studies in a sub-set of patients to assess treatment effects on basal forebrain atrophy. With the limited number of therapeutic clinical trials that had been conducted in DLB at the time the AscenD-LB trial was initiated, there was limited precedence for clinical endpoints that could detect treatment effects, particularly for treatments targeting the underlying disease process. The NTB that was the primary outcome measure in AscenD-LB was patterned after a cognitive test battery in a trial of rivastigmine (22), one of the few published trials in DLB that had shown a positive effect on the attentional deficit that is a prominent component of the dementia of DLB. However, in AscenD-LB both the CDR-SB and TUG performed consistently better than the NTB. As all patients were receiving cholinesterase inhibitors, and cholinesterase inhibitors can improve cognition but generally provide no benefit to motor function (23), one explanation for the relative lack of effects on the cognitive endpoints (relative to that seen on the TUG and the CDR-SB) is that the cholinesterase inhibitors had provided much of the cognitive benefit possible in the study, which limited the ability to demonstrate a cognitive effect, but did not impact the ability to demonstrate motor function effects on the TUG test, nor on the functional components of the CDR-SB (e.g. on personal care). In addition, the minimal executive function deficits at baseline will have limited the ability to improve outcomes on the components of the NTB that were assessing executive function (i.e., a ceiling effect). While this may be attributed to the combination of the ceiling effect imposed by the patients in the study receiving a cholinesterase inhibitor and the modest deficits in the tests of executive function at baseline, the CDR-SB and TUG by being able to capture treatment effects on both cognitive and motor aspects of the disease may be intrinsically better suited as endpoints for disease modifying approaches for DLB. Further supporting the use of these endpoints to assess effects on the underlying disease process, in longitudinal studies progression of cortical atrophy by MRI has been correlated to the CDR-SB in patients with DLB (24), and progression of basal forebrain atrophy to gait dysfunction in PD (25). In addition, clinical disease progression was well captured by the CDR-SB in a large longitudinal cohort (26). Finally, the CDR-SB through being primarily dependent on caregiver report and based on assessment of cognition and function over the two prior to assessment (i.e., intended to provide an assessment integrated over two weeks), should be less impacted by the cognitive fluctuations that are common in DLB. These fluctuations have the potential to be a relevant noise factor in a single time point assessment such as the NTB.

Potential reasons, other than consensus on clinical endpoints, for the lack of drug development for DLB include (1) the difficulty to distinguish DLB from AD and (2) heterogeneity in terms of rate of disease progression. On the former, recent literature suggests that the current consensus criteria combined with a pre-mortem abnormal DaTscan has 86% diagnostic accuracy, including a specificity of 92%, for differentiating autopsy confirmed DLB from autopsy confirmed AD (27). This is in line with our finding that it was possible to identify and enroll a DLB patient population with a profile consistent with DLB, including prominent attentional deficits, in the AscenD-LB study. With regard to heterogeneity in progression, natural history studies suggest that much of the heterogeneity in DLB is related to AD co-pathology (28). Accordingly, the exclusion of patients with AD co-pathology (assessed by plasma ptau181 at study entry) should lead to a more homogenous patient population. Indeed, in AscenD-LB the neflamapimod treatment effects were more consistent and of greater magnitude in the patients without AD co-pathology (i.e., patients with pure DLB). This concept is further supported by recently reported results (29) with the PDE9 inhibitor, irsenontrine; in a 12-week placebo-controlled phase 2 study in DLB, where “amyloid negative” patients (evidenced by a high plasma Aβ42/40 ratio) demonstrated a trend (p=0.053) towards improvement in cognition, measured using the Montreal Cognitive Assessment, while no such trends were evident in “amyloid positive” patients. Of note, because plasma ptau181 is highly correlated to elevation in brain amyloid by PET scan (30) the exclusion of patients with elevated plasma ptau181 should by itself increase the specificity of the inclusion criteria for DLB versus AD.

While showing limited efficacy over 16 weeks in DLB patients with AD co-pathology, our findings do not preclude the possibility that a treatment of longer duration would demonstrate effects on disease progression in these patients. Indeed, there are some positive efficacy trends in these patients and if AD co-pathology and neurodegeneration were dependent on progression of basal forebrain degeneration, then an agent, such as neflamapimod, targeting the basal forebrain pathogenic process would slow further progression of the neurodegenerative process in DLB patients with established AD co-pathology. In the AD field, though the topic continues to be debated, increasing evidence points to the basal forebrain as being a driver of development of both amyloid pathology and neurodegeneration in the cortex (31, 32). In DLB as well, basal forebrain atrophy appears to be a major driver of cortical neurodegeneration (19, 33), though independent effects of amyloid pathology may also contribute (30). Moreover, in a recent analysis the CSF proteome in DLB with amyloid pathology was different from those in AD dementia and pure DLB indicating “that amyloid pathology in DLB is not an independent co-pathology” (34).

Slowing on the EEG over posterior regions of the brain has been proposed as a biomarker of disease progression in DLB (19). Though perhaps limited by the number of patients who were able to obtain EEGs in the study, an effect on reversing this slowing was not seen in AscenD-LB. The lack of such effect otherwise is potentially due to all the patients in the were receiving cholinesterase inhibitors and that therapy is known to have positive effects on slowing on EEG, i.e., as with the NTB, there may have been ceiling effects with respect to EEG slowing. The potential EEG effect that was seen in AscenD-LB was improvement with neflamapimod treatment compared to placebo in beta band functional connectivity, an effect that may be particularly relevant because it has been reported that deficits in beta band functional connectivity best differentiates DLB from AD on the EEG (35). Accordingly, EEGs will be obtained in the phase 2b study and beta band functional connectivity will be the primary outcome measure with respect to the EEG. In addition, Eyes-Closed-to-Eyes Open alpha reactivity, which was not evaluated in AscenD-LB, has been included as secondary EEG endpoint in AscenD-LB because a report (36) published after the study was conducted indicate that alpha reactivity is a specific measure for DLB and a measure of “cholinergic integrity” in both DLB and AD. To further evaluate potential effects on the underlying disease process, structural and functional MRI will be evaluated in 40 patients to assess treatment effects on atrophy of the basal forebrain, as well its functional connectivity.

In summary, the AscenD-LB phase 2a clinical trial demonstrated the feasibility of conducting a clinical trial with a potentially disease-modifying agent in patients with DLB and obtaining preliminary evidence of clinical efficacy. The results and major learnings with respect to the performance of various clinical and biomarker endpoints have informed on the design of a phase 2b clinical trial, the RewinD-LB study, whose broad objective is to replicate the phase 2a results and a primary objective of confirming the effects on the CDR-SB. With success, the RewinD-LB study would provide definitive proof-of-concept for neflamapimod as a treatment for DLB and further validate the pathogenic model that provided the mechanistic rationale for targeting the cholinergic deficit in DLB with neflamapimod.

## Notes

*Ethical standards:* All three clinical trials conducted in accordance with Good Clinical Practice guidelines and the Declaration of Helsinki. Applicable local/ central ethics committee or IRB approvals were obtained. All participants provided written informed consent and were not compensated.
